# Detection of genetically modified organisms (GMOs) using isothermal amplification of target DNA sequences

**DOI:** 10.1186/1472-6750-9-7

**Published:** 2009-02-02

**Authors:** David Lee, Maurizio La Mura, Theo R Allnutt, Wayne Powell

**Affiliations:** 1 John Bingham Laboratory, National Institute of Agricultural Botany, Huntingdon Road, Cambridge, UK; 2 Dipartimento di Scienze del Suolo, della Pianta, dell' Ambiente e delle Produzioni Animali, University of Naples, Federico II, Via Universita 100, 80055, Portici, Italy; 3 Central Science Laboratory, Sand Hutton, York, UK; 4 Institute of Biological, Environmental & Rural Sciences (IBERS), Aberystwyth University, Penglais, Aberystwyth, Ceredigion, UK

## Abstract

**Background:**

The most common method of GMO detection is based upon the amplification of GMO-specific DNA amplicons using the polymerase chain reaction (PCR). Here we have applied the loop-mediated isothermal amplification (LAMP) method to amplify GMO-related DNA sequences, 'internal' commonly-used motifs for controlling transgene expression and event-specific (plant-transgene) junctions.

**Results:**

We have tested the specificity and sensitivity of the technique for use in GMO studies. Results show that detection of 0.01% GMO in equivalent background DNA was possible and dilutions of template suggest that detection from single copies of the template may be possible using LAMP.

**Conclusion:**

This work shows that GMO detection can be carried out using LAMP for routine screening as well as for specific events detection. Moreover, the sensitivity and ability to amplify targets, even with a high background of DNA, here demonstrated, highlights the advantages of this isothermal amplification when applied for GMO detection.

## Background

The ability to detect the presence of GMO is pivotal for consumers to be able to exercise their lifestyle choice of whether to consume, or not, food containing GMOs. Though the detection and quantification of GMO proteins using immunoassay has been reported [1], denaturation of the protein during processing makes the method unsuitable for GMO testing and quantification of food. The durability of DNA makes it a better substrate for testing and its amplification by PCR is the method of choice, as recommended by the EC (2004/787), for detection and quantification of GMO in samples.

An alternative DNA amplification method was described by Notomi and coworkers [2] called 'loop mediated isothermal amplification' (LAMP). The LAMP assay relies on the design of a set of primers that generate stem looped (hairpin) structures during the early stage of DNA synthesis. The hairpin structures form because two of the primers used contain, at their 5' end, a reverse complement of a sequence that is present in the target further downstream of the initial binding site. Displacement primers help the formation of these hairpins at the ends of the DNA strands and once formed, these structures can be copied into a series of DNA fragments containing multiple units of the target sequence under isothermal conditions utilizing the displacement properties of *Bst *polymerase (see [3]). Although LAMP was first described using a set of four primers, enhanced sensitivity was reported using an additional pair of loop primers [4]. As the reactions are performed at a single temperature, LAMP assays can be performed very quickly since there are no separate denaturation, annealing and extension steps, and as such, reactions do not require thermalcyclers. Here we assess the LAMP protocol for the detection of GMOs using primers that target event-specific sequences for transgenic MS8 and RF3 oilseed rape (*Brassica napus *L.) and generic GMO sequences such as the cauliflower mosaic virus 35S promoter (*P-35S*) and the promoter and terminator for the nopaline synthase gene (*P-nos *and *T-nos*, respectively) from *Agrobacterium *spp.

## Results and discussion

### Specificty of LAMP

The LAMP technique relies on the design of an inter-related set of primers. The orientation and positions are important for self-priming through stem-looped products that drive and perpetuate the reaction.

The OSR events MS8 and RF3 arise from the insertion of two closely related transgenes from the plasmids, pTHW107 and pTHW118, respectively [5]. The former encodes the Barnase gene that give rise to male sterility, which is replaced in the latter by the Barstar gene which restores fertility: both also have the selectable marker *bar *which confers tolerance to the herbicide glufosinate ammonium. Though the left border of the RF3 insert has undergone rearrangement in the form of duplication and inversion [5], the right borders of both events are relatively intact (our data [6] which agree with the two sequences in the database).

**Figure 1:**
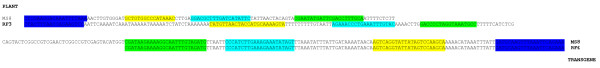
**Right border sequences of MS8 and RF3**. Sequences of the plant (above) and transgene (below) at the right border junctions for MS8 and RF3. Highlighted sequences are the targets of the LAMP primers. The plant sequences are those shown in Table 1: dark blue bases highlighted are the outer displacement primers, yellow and green sequences are the 5' and 3' ends of the LAMP primers respectively, and light blue sequences depict the loop primers.

**Figure 2:**
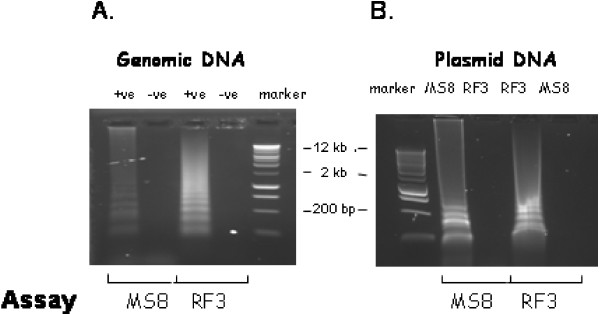
**Detection of MS8 and RF3 transgene demonstrate specificity of LAMP**. Testing of assays on MS8/RF3 oilseed rape DNA (A) and separately using plasmids (B) to demonstrate the specificity of the reactions. +ve and -ve denote the presence or absence of target DNA, respectively. Sizes of marker bands are shown.

Even though the insertions of the transgenes have different breakpoints from the plasmids, they are very close so it was possible to design assays for the RF3 and MS8 events utilizing a common set of primers within the transgenes (Figure 1). When used in conjunction with primers for the plant sequence at the border of each event, they are able to detect each event (Figure 2). Since they have 50% of primers in common, it was important to determine whether there was any cross reaction between the assays. Specificity of the two assays was tested using plasmid DNA for each event. No cross-reaction between the two targets was observed (Figure 2).

### LAMP sensitivity

**Figure 3:**
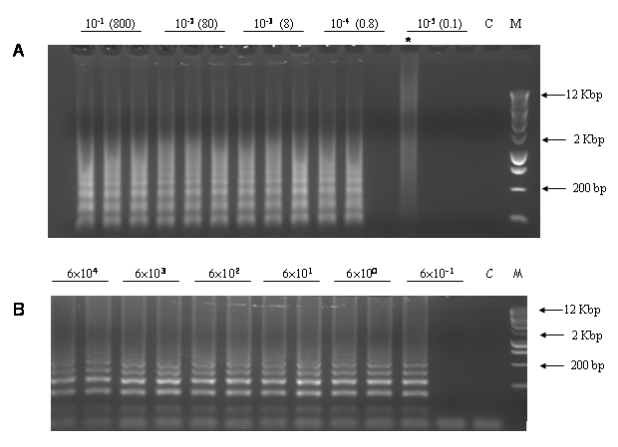
**Sensitivity assessment of LAMP Detection**. A. Sensitivity of LAMP using genomic target. DNA from MS8/RF3 sample (16 ng.μL^-1^) was serially diluted and amplified by LAMP, in triplicate, using primers to assay for the RF3 junction. The numbers in parenthesis represent the approximate copy numbers of the target assuming that the sample represents RF3 in a hemizygous state (determined using RT-PCR data not shown) for the transgene and using 1 pg as the genome size for oilseed rape. C is the no DNA control and M represents molecular size markers. The smear (*) shows an example of non-specific amplification. B. Sensitivity of LAMP using plasmid target. Serial dilutions of the plasmid pGreenII were amplified using primers for the Pnos target. Numbers represent the calculated copy numbers of the plasmid derived from the DNA value. C is the no DNA control and M represents molecular sized markers.

The sensitivity of the LAMP reaction was assessed in two ways: copy number detection and background in which 10 copies of the target could be detected. Copy number detection was measured by serial dilutions of known amounts of DNA containing the target sequences, either as genomic or plasmid DNA (Figure 3). Reactions fail in both assays at DNA molecule number of less than 1 which is consistent with the stochastic probability of a target being present [7].

**Figure 4:**
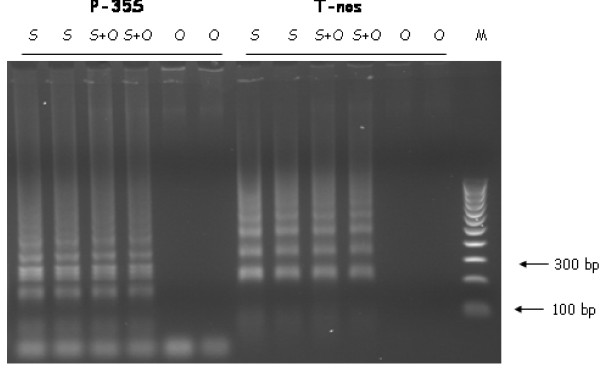
**Sensitivity of LAMP reactions with background DNA**. LAMP of samples containing 10 copies of RoundUp ReadyTM soya transgene (S), and in a background of 100 ng of oilseed rape DNA (O). M is molecular size standard.

We note that sometimes non-specific amplification can also take place, especially where the target DNA is absent (see Figure 3) and there is low amounts of DNA present in the reaction (cf Figures 3a and 4). These do not form the specific banding patterns representing the different multimeric LAMP products that are characteristic for each assay and thus can be easily distinguished on a gel. Alternative banding patterns have been observed, also for low template reactions; analyses of these products show that they are formed by interactions of the primers used [8], to form LAMP equivalents of primer-dimers. Two factors seem to be important: in the absence of target, low background DNA may aid the formation of non-specific products that go on to be amplified; and freeze-thaw repetitions may induce damage to the primers to permit the formation of 'primer intermediates' which can then be amplified. Since LAMP is capable of non-specific amplification, techniques that rely on the detection of by-products of DNA synthesis, e.g., the use of magnesium pyrophosphate precipitation [9] or the use of SyBr Green dye [10] may not be able to distinguish between real and false positives. LAMP can still be useful as a quick primary gel-free screen, where 'positives' can then undergo a second screening such as gel electrophoresis.

The sensitivity of the LAMP assay and its suitability for practical GMO detection was tested using assays for commonly-used sequence motifs, the CaMV 35S promoter (*P-35S*) and the *Agrobacterium *promoter and terminator for the nopaline synthase gene (*P-nos *and *T-nos*, respectively). These sequences are commonly used in constructs used to create approved GM events (see [11]). RoundUp Ready™ soya construct contains both *P-35S *and *T-nos *so provided convenient template for testing the assays. We used a sample where the copy number of the GM has been well characterized and thus control the number of template in each reaction.

LAMP sensitivity was assessed by the detection of ten RoundUp Ready™ GMO targets in a background of 100 ng of genomic oilseed rape DNA (Figure 3). Since the C value of both species is approximately 1 pg [12], this background DNA represents a GMO level of 0.01% for both *T-nos *and *P-35S *assays. OSR DNA was used because we did not have any soya DNA free from RoundUp Ready™. DNA extracted from our 0% CRM was shown to contain 0.002% GMO [13]. The use of 100 ng of this sample would be equivalent to adding 200 copies of the transgene sequence, considerably more than the experimental input of 10 copies. We believe the use of OSR DNA to be a valid substitute since none of the primer sequences for either assay will be present in non-transgenic soya or oilseed rape.

We have tested the upper limits of DNA that LAMP reactions can tolerate and found that up to 200 ng DNA in a 20 μl reaction, positive detection is reproducible. Above this DNA level, reactions become unreliable (data not shown).

We have found that denaturation of template was a pre-requisite step prior to amplification, unlike results found by Nagamine and co-workers [14]. This can be explained by the fact that we do not use a base pair destabiliser, such as betaine, in the reaction buffer. Since we are detecting down to near single copies of templates, our results suggest no benefit to the sensitivity of the assay by their inclusion. The consistent amplification within all dilutions showed that LAMP is an 'all or nothing' reaction, with little of the tailing off effect that is often observed in PCRs with diluting templates. This makes it easy to identify positive reactions. Together with specificity and the speed at which reactions can be performed, LAMP is an excellent method for diagnostics [8,10,15].

The use of CaMV 35S promoter sequence in LAMP has previously been reported as a screening method [16]. Here we demonstrate the sensitivity and reliability of the LAMP method for GMO detection, both with generic and GMO-specific assays. The ability to perform reactions in a simple heated block or water bath without the need for thermal cycling makes testing using LAMP more accessible.

## Conclusion

That LAMP is able to detect very small amounts of target and do that even in high amounts of background DNA makes it ideal for GMO detection. GMO testing can be performed in steps: routine screening for the presence of GMOs using generic assays such as for 35S promoters and T-nos; and if required, identification of specific events can be performed using event-specific assays. Equally, direct screening using event-specific assays is also feasible. The levels of sensitivity are orders of magnitude below the permissible threshold for GMO in food and feed (EC regulation 1830/2003), ensuring the detection of the presence of GMOs at acceptable levels and the reliable detection of any presence of unauthorised GM events, for which at present there is no legally acceptable lower limit (according to EC regulations).

## Methods

### Plant material

Conventional oilseed rape (OSR) seed, variety 'Hearty' was a gift from Christine Lewis, NIAB. The sample was originally purchased from Monsanto UK Ltd (Cambridge, UK). DNA was extracted by grinding 2 g seed with 10 ml extraction buffer [0.5 M NaCl, 0.1 M EDTA pH 8 and 1% (w/v) SDS] in a mortar with a pestle. The sample was emulsified with 5 ml of chloroform:isoamyl alcohol (24:1) and poured into a 20 ml Falcon polypropylene tube. After centrifugation at 1000 g for 5 mins, the aqueous phase was transferred to a new tube and nucleic acids were precipitated by the addition of an equal volume of isopropanol and collected by centrifugation at 750 g for 5 mins. The pellet was resuspended in 1 ml of 10 × TE (100 mM Tris:Cl, 10 mM EDTA pH 8) and further purified using Qiagen DNeasy kit to a final volume of 200 μL of 0.1 × TE (1 mM Tris 0.1 mM EDTA pH 8). The DNA concentration was determined by titration in an agarose gel against dilutions of a commercial lambda sample (BRL, Invitrogen, Paisley, UK).

DNA from the oilseed rape MS8/RF3 was extracted from seedlings from a selfed MS8/RF3 plant [17] using DNeasy Plant DNA Extraction Kit (QIAGEN, Crawley, UK). The parent plant was genotyped to be MS8MS8/RF3rf3 using real-time PCR (data not shown). The sample was quantified using picogreen fluorescence (Molecular Probes Inc., Invitrogen). DNA containing RoundUp Ready™ soya was extracted from Soya Roundup Ready™ GMO Reference Material (Fluka Biochemika, Sigma-Aldrich, Dorset, UK) and the GMO concentration of the sample has been accurately determined by dilutions of template combined with statistical analysis [13].

### Plasmid DNA

The plasmid pGreenII 0049 was a gift from Mark Smedley and Wendy Harwood of the John Innes Centre. Details of the plasmid can be found at the website: 

Plasmids containing each event were constructed to test the specificity of the MS8 and RF3 assays separately. The junction at the right borders of the transgenes were amplified by PCR from the MS8/RF3 DNA sample using the displacement (outer) primers of the LAMP reactions, MS8-RF3 DisplR (B3c) separately with MS8 DisplF (F3) and RF3 DisplF (F3), to amplify the MS8 and RF3 junctions, respectively (see Figure 1 and Table 1). The fragments were cloned into pGEM-T vector (Promega, Southampton, UK) and transformed into DH5α. Clones containing the correct inserts were confirmed by sequencing.

**Table 1:** Primers used in this study.

Primer name	Sequence (5'-3')
	
MS8 LampF (F1c-F2)	TGCAAAGGTCGAATCATATTCG-TTTT-GCTGTGGCCCATAAC
MS8 LoopF (LoopFc)	GAATATGATCAAAGCGTCC
MS8 DisplF (F3)	CTCGAAGGACAAATTTTAAA
	
MS8-RF3 LampR (B1-B2c)	CGATAAGAAAAGGCAATTTGTAGATG-TTTT-GCTTGGACTATAATACCTGACT
MS8-RF3 LoopR (LoopB)	CCCATCTTGAAAGAAATATAGT
MS8-RF3 DisplR (B3c)	TTCTGAATTTAAACTTGCATC
	
RF3 LampF (F1c-F2)	GGCATTTACCTAGGGGTC-TTTT-ATGTTAACTACCATGCAAAAGTA
RF3 LoopF (LoopFc)	GTACAAATTTCAGGGTTTCT
RF3 DisplF (F3)	CTACTTTAACCAGAAGTCC
	
T-nos LampF (F1c-F2)	AGATGGTTTTTATGATTAG-TTTT-ATTTATCCTAGTTTGCGC
T-nos LoopF (LoopFc	CAATTATACATTTAATACGCG
T-nos DisplF (F3)	CATAGATGACACCGCG
T-nos LampR (B1-B2c)	TAATTCAACAGAATTATATG-TTTT-AAGTTTCTTAAGATTGAATCCTG
T-nos LoopR (LoopB)	TGCAAGACCGGC
T-nos DisplR (B3c)	GATCGTTCAAACATTTGG
	
CaMV35P LampF (F1c-F2)	GTCTTCAAAGCAAGTGG-TTTT-GGATAGTGGGATTGTGCG
CaMV35P LoopF (LoopFc)	TCCACTGACGTAAGGG
CaMV35P DisplF (F3)	AGGAAGGGTCTTGCG
CaMV35P LampR (B1-B2c)	TTCCACGAT GCTCCTCG-TTTT-CCTCTGCCGACAGTGG
CaMV35P LoopR (LoopB)	GGGGTCCATCTTTGGG
CaMV35P DisplR (B3c)	ATAAAGGAAAGGCCATCG
	
P-nos LampF (F1c-F2)	TGCGCGTTCAAAAGTCG-TTTT-ATTTATGGAACGTCAGTGG
P-nos LoopF (LoopFc)	AGCTAGCAAATATTTCTTG
P-nos DisplF (F3)	TAATTGGATACCGAGG
P-nos LampR (B1-B2c)	TGACGTATG TGCTTAGCTC-TTTT-AACCGCAACGTTGAAG
P-nos LoopR (LoopB)	TAAACTCCAGAAACCC
P-nos DisplR (B3c)	ACAAGCCGTTTTACG

Primer names and sequences used are listed. Nomenclature used by Nagamine *et al*. (2002) is shown in parentheses.

### Sub-heading for this section

#### Assay Design

Target sequences for *P-35S*, *P-nos *and *T-nos *were chosen based upon common identity between different plasmids in the EMBL database containing the promoters and terminator. The sequences of the targets and positions of the primers are provided (see Additional file 1).

Primers for each target segment have Tm's of 50–52°C (calculated using the 2 × AT, 4 × GC formula), except for the F2 and B2 regions (3' of the LAMP primers), where the Tm was 54–56°C. Primer sequences are listed in Table 1.

#### LAMP reactions

For LAMP reactions, primers were purchased from Sigma-genosys (Table 1). Reactions were performed in 20 μl containing 1 × Bst pol buffer (NEB, Ipswich, UK) with 0.4 mM each dNTP with the appropriate primers listed in Table 1: Displacement primers were each used at a concentration of 0.2 μM; Loop primers at 0.4 μM and LAMP primers at 0.8 μM in the reactions. Enough reaction reagents, without template and enzyme, were mixed together and split into two. Template (1 μl) was added to 9 μl of the mix and the samples denatured at 95°C for 2 mins and then cooled to 4°C. *Bst *pol was added to the remaining reaction mix to a concentration of 3.2 U.μl^-1^, mixed thoroughly, and 10 μl was added to each reaction. Reactions were incubated at 55°C for 2 hours, followed by 80°C for 10 mins to inactivate the enzyme and stored at 4°C until analysed. Aliquots of the reactions (5 μl) were run on 1.5% (w/v) agarose gels, containing ethidium bromide (0.05 ug/ml), in 0.5 × TBE buffer [0.045 M tris-borate; 0.001 M EDTA (pH8)] at 20 V/cm for 1 hour. DNA was visualized by UV illumination.

## Authors' contributions

DL, as project leader, coordinated the work and wrote the manuscript. MLM performed lab work. TA provided data and material for the experiments. WP, as Group Leader provided focus and direction. All authors contributed to discussions, read and approved the final manuscript.

## Supplementary Material

Additional file 1 **Sequences of LAMP targets.** The sequences of the different genetic elements used to design the LAMP assays are shown together with a genebank accession number that contains the sequence. The sequences targeted by the LAMP primers are coloured. Click here for file

## References

[B1] Lipp M, Anklam E, Stave JW (2000). Validation of an immunoassay for detection and quantitation of a genetically modified soybean in food and food fractions using reference materials: interlaboratory study. J AOAC Int.

[B2] Notomi T, Okayama H, Masubuchi H, Yonekawa T, Watanabe K, Amino N, Hase T (2000). Loop-mediated isothermal amplification of DNA. Nucl Acids Res.

[B3] Loop animation. Eiken Genome Site.

[B4] Nagamine K, Hase T, Notomi T (2002). Accelerated reaction by loop-mediated isothermal amplification using loop primers. Mol Cell Probes.

[B5] Report on the molecular characterisation of the genetic map of event Ms8 × Rf3. http://www.biosafety.be/gmcropff/EN/TP/MGC_reports/Report_Ms8xRf3.pdf.

[B6] Plasmid standards for real time PCR and GM enforcement testing. http://www.gm-inspectorate.gov.uk/documents/PLASMID.pdf.

[B7] Kay S, Eede G van den (2001). The limits of GMO detection. Nature Biotechnology.

[B8] Kuboki N, Inoue N, Sakurai T, Di Cello F, Grab DJ, Suzuki H, Sugimoto C, Igarashi I (2003). Loop-mediated isothermal amplification for detection of African trypanosomes. J Clin Microbiol.

[B9] Mori Y, Nagamine K, Tomita N, Notomi T (2001). Detection of loop mediated isothermal amplification reaction by turbidity derived from magnesium pyrophosphate formation. Biochem Biophys Res Commun.

[B10] Iwamoto T, Sonobe T, Hayashi K (2003). Loop-mediated isothermal amplification for direct detection of *Mycobacterium tuberculosis *complex, *M. avium*, and *M. intracellulare *in sputum samples. J Clin Microbiol.

[B11] Genetically Modified (GM) Crops: molecular and regulatory details. http://www.bats.ch/gmo-watch/GVO-report140703.pdf.

[B12] Arumuganathan K, Earle ED (1991). Nuclear DNA content of some important plant species. Table I (nuclear DNA content of a number of important plant species as determined by flow cytometry). Plant Molecular Biology Reporter.

[B13] Lee D, La Mura M, Greenland A, Mackay I (2008). Quantitation using informative zeros (QUIZ): application for GMO detection and quantification without recourse to certified reference material. Food Chemistry.

[B14] Nagamine K, Watanabe K, Ohtsuka K, Hase T, Tsugunori T (2001). Loop-mediated isothermal amplification reactions using a nondenatured template. Clin Chem.

[B15] Thai HTC, Le MQ, Vuong CD, Parida M, Minekawa H, Notomi T, Hasebe F, Morita K (2004). Development and evaluation of a novel loop-mediated isothermal amplification method for rapid detection of severe acute respiratory syndrome coronavirus. J Clin Microbiol.

[B16] Fukuta S, Mizukami Y, Ishida A, Ueda J, Hasegawa M, Hayashi I, Hashimoto M, Kanbe M (2004). Real-time loop-mediated isothermal amplification for the CaMV-35S promoter as a screening method for genetically modified organisms. Eur Food Res Technol.

[B17] Weekes R, Deppe C, Allnutt T, Boffey C, Morgan D, Morgan S, Bilton M, Daniels R, Henry C (2005). Crop-to-crop gene flow using farm scale sites of oilseed rape (*Brassica napus*) in the UK. Transgenic Res.

