# Supplementary material for: Detection of genetically modified organisms (GMOs) using isothermal amplification of target DNA sequences
Source: BMC Biotechnol. 2009 Feb 2;9:7. doi: 10.1186/1472-6750-9-7 (PMC2656497; doi:10.1186/1472-6750-9-7)
Supplement: Additional file 1 — Sequences of LAMP targets. The sequences of the different genetic elements used to design the LAMP assays are shown together with a genebank accession number that contains the sequence. The sequences targeted by the LAMP primers are coloured. [file 1472-6750-9-7-S1.doc]

**Tnos**

[gi:193227101]

catagatgacaccgcgcgcgataatttatcctagtttgcgcgctatattttgttttctatcgcgtattaaatgtataattgcgggactctaatcataaaaacccatctcataaataacgtcatgcattacatgttaattattacatgcttaacgtaattcaacagaaattatatgataatcatcgcaagaccggcaacaggattcaatcttaagaaactttattgccaaatgtttgaacgatc

B3: catagatgacaccgcg

B1+B2: agatgggtttttatgattag-TTTT-atttatcctagtttgcgc

LoopB: caattatacatttaatacgcg

F3: gatcgttcaaacatttgg

F1+F2: taattcaacagaaattatatg-TTTT-aagtttcttaagattgaatcctg

LoopF: tcgcaagaccggc

**Pnos**

[gi:163961140]

taattggataccgaggggaatttatggaacgtcagtggagcatttttgacaagaaatatttgctagctgatagtgaccttaggcgacttttgaacgcgcaataatggtttctgacgtatgtgcttagctcattaaactccagaaacccgcggctgagtggctccttcaacgttgcggttctgtcagttccaaacgtaaaacggcttgt

B3: taattggataccgagg

B1+B2: tgcgcgttcaaaagtcg-TTTT-atttatggaacgtcagtgg

LoopB: agctagcaaatatttcttg

F3: acaagccgttttacg

F1+F2: tgacgtatgtgcttagctc–TTTT-aaccgcaacgttgaag

LoopF: taaactccagaaaccc

**CaMV 35S Promoter**

[gi:190887195]

aggaagggtcttgcgaaggatagtgggattgtgcgtcatcccttacgtcagtggagatatcacatcaatccacttgctttgaagacgtggttggaacgtcttctttttccacgatgctcctcgtgggtgggggtccatctttgggaccactgtcggcagaggcatcttcaacgatggcctttcctttat

F3: aggaagggtcttgcg

F1+F2: gtcttcaaagcaagtgg-TTTT-ggatagtgggattgtgcg

loopF: tccactgacgtaaggg

B3: ataaaggaaaggccatcg

B2+B1: ttccacgat gctcctcg-TTTT-cctctgccgacagtgg

LoopB: ggggtccatctttggg

**Additional file 1 – sequences of LAMP targets**
